# Effects of High-Speed Shearing Treatment on the Physical Properties of Carbohydrate-Binder Mixture during Gelatinization for Preparing Freeze-Dried Soup Products

**DOI:** 10.3390/foods13172661

**Published:** 2024-08-23

**Authors:** Ga-Yang Lee, Min-Jeong Jung, Byoung-Mok Kim, Ha Ram Kim, Joon-Young Jun, Nam Hee Kim

**Affiliations:** 1 Food Convergence Research Division, Korea Food Research Institute, Wanju 55365, Republic of Korea; rkdid0925@gmail.com (G.-Y.L.); mjjung@kfri.re.kr (M.-J.J.); bmkim@kfri.re.kr (B.-M.K.); haram.kim@kfri.re.kr (H.R.K.); 2 N Kim lab, DAESAN Inc., Gangneung Science & Industry Promotion Agency, Gangneung 25440, Republic of Korea

**Keywords:** home meal replacement food, freeze-dried soup, carbohydrate-binder, high-speed shearing homogenization, hydrothermal gelatinization

## Abstract

Modernization has led to a large convenience food market, and the demand for freeze-dried (FD) soup products is increasing in the Republic of Korea. FD soup products are easy to eat without cooking and can be stored for long periods. However, it is often difficult to ensure sensory satisfaction after rehydration of FD soup products; in particular, the ingredients are not evenly dispersed. Therefore, a stable dispersion or reconstitution of the FD soup products is required after rehydration. Here, the effects of high-speed shearing homogenization on the physical properties of a carbohydrate-binder mixture comprising maltodextrin, potato starch, and rice flour were investigated during hydrothermal gelatinization. To find a suitable treatment condition, different homogenization eras, speeds, and concentrations of the binder mixture were considered; in particular, the homogenization eras were set by considering the hydrothermal property of the binder mixture profiled using differential scanning calorimetry. The viscosity of the binder mixture and the compression strength and microstructure of the FD binder block, including the dispersion stability after rehydration, were evaluated. The quality of the FD binder block was improved by homogenization above 5000 rpm when the core temperature of the binder mixture reached approximately *T_o_* at 14.5–21.8% concentrations. The improved FD binder block exhibited a fine surface and tiny porous microstructure compared with the control (with continuous agitation at 250 rpm). The control block was divided into two phases, whereas the improved block maintained the initial dispersion stability at 50 °C for 1 h. These results are expected to be referenced for the purpose of improving the quality of the FD soup products.

## 1. Introduction

Rapid modernization has led to significant convenience in the food market. Home meal replacement (HMR) is a convenience food that refers to a one-dish meal, which can be consumed both at home and in stores, does not require consumers to go through a cumbersome cooking process, and can simply be heated before eating [1]. According to the Food Information Statistics System of the Korea Agro-Fisheries & Food Trade Corporation [2], the HMR market size in the Republic of Korea was approximately USD 3.2 billion in 2021, of which convenience soup (including broth and stew) products accounted for approximately 18.8%; various Korean soups, such as doenjang soup, chicken soup, and yukgaejang, are manufactured and distributed as convenience soup products.

Freeze-drying is often used to produce soup products and prevent food deterioration during long-term transport. In the freeze-drying process, the water in food is immediately sublimated from solid to gas in a vacuum; thus, freeze-dried (FD) food materials can easily return to their original state with quick rehydration and/or reconstitution, with minimal food quality loss [3,4]. In addition, freeze-drying is a good way to extend the shelf life of liquid-based commercial products such as soups, broths, and stews [5]. These products are generally made by combining different ingredients, such as vegetables, poultry, meat, or seafood, and consumers are satisfied only when the product has a complete shape when opened, and all ingredients are in harmony when consumed [6].

However, it is often difficult to ensure sensory satisfaction after rehydration in FD soup products. As solid ingredients tend to settle at the bottom because of their heavy mass, not all ingredients of the product are dispersed. Therefore, a stable dispersion or reconstitution of FD soup products is required after rehydration during product development. Carbohydrates (e.g., dextrin, starch, rice flour, pectin, and gums) are commonly used as binders and/or solidifiers in the preparation of FD soup products because they bind raw and subsidiary materials together and form spaces for moisture to penetrate, while maintaining a precast shape [7]. The coverage, types, and physicochemical properties of the carbohydrate-binder of FD products can significantly affect the stability of the final product, which has long been considered an important factor in powdering technology associated with the rehydration behavior of dried foods [5,8]. In general, the preparation process of block-type FD soup products includes pretreatment of raw materials, hydrothermal mixing of the binder, source base, major and minor ingredients in that order, heat treatment, cooling and molding, rapid freezing, freeze-drying, mold removal, and packaging [9]. During hydrothermal mixing, gelatinization of the carbohydrate-binder and cooking of ingredients are induced so that consumers can consume it without additional cooking.

Several researchers have examined the performance of freeze-drying in terms of changes in the physicochemical properties of food materials [6,10], industrial applicability [11,12], and technical advancement [4,13]. However, few studies have been conducted on the rehydration or reconstitution performance of FD soup products [7,14]. The gelatinization and retrogradation of starch during drying may cause an undesirable decrease in the viscosity of soup containing starch when rehydrated [15]. Physical treatments such as annealing, heat-moisture treatment, and homogenization can alter the physicochemical properties of starches [16].

In this study, a carbohydrate-binder mixture composed of maltodextrin, potato starch, and rice flour was treated with a high-speed shear homogenizer during hydrothermal gelatinization, and the effects on the physical properties of the mixture and its FD block were investigated. To find a suitable treatment condition, different homogenization eras, speeds, and concentrations of the mixture were considered in the order mentioned. The viscosity of the binder mixture, compression strength, and microstructure of the FD block, including the dispersion stability after rehydration, were evaluated from the perspective of industrial applications of block-type FD soup products.

## 2. Materials and Methods

### 2.1. Materials

Maltodextrin (DE 28, Daesang Co., Seoul, Republic of Korea), potato starch (Seoryong Industrial Food Co. Ltd., Seogwipo, Republic of Korea), and rice flour (SaeRom Food Agricultural Co., Icheon, Republic of Korea) were used as carbohydrate-binders for preparing an FD soup base, which was food-grade.

### 2.2. Determination of Differential Scanning Calorimetry

The thermal properties of the carbohydrate-binders were profiled using differential scanning calorimetry (DSC; DSC 4000, Perkin Elmer, Waltham, MA, USA) following the method recommended by Liu et al. [17], with slight modifications. The samples were weighed in a stainless-steel large-volume pan at a 1:3 ratio (*w*:*w*). The pan was hermetically sealed and stored at room temperature overnight to maintain moisture equilibrium. The thermodynamic curve scan was conducted over a temperature range of 30–150 °C at an increasing rate of 10 °C/min, with an empty pan as a reference. The onset (*T_o_*), peak (*T_p_*), and conclusion (*T_c_*) temperatures and enthalpy change (Δ*H*) were calculated using Pyris software V. 13.0 (Perkin Elmer, USA).

### 2.3. Preparation of Carbohydrate-Binder Mixture

The mixing composition of carbohydrate-binders followed the base recipe for the preparation of typical Korean soups used by an FD soup product manufacturer (Donglimfood Inc., Gangneung, Republic of Korea), which was composed of 10 g maltodextrin, 2.5 g potato starch, and 2.0 g rice flour in 100 mL water (totally 14.5%, *w*/*v*).

### 2.4. Experimental Design for Physical Treatment

To find a suitable homogenization condition, the effects of the homogenization era, speed, and concentration of the carbohydrate-binder mixture on the viscosity of the carbohydrate-binder mixture and compression strength of the FD binder blocks were investigated. The homogenization era was set by considering the thermal properties [onset (*T_o_*), peak (*T_p_*), and conclusion (*T_c_*) temperatures] of the carbohydrate-binder mixtures during gelatinization.

The experimental conditions are listed in Table 1 and described as follows: (1) continuous agitation at 25 °C (NH); (2) continuous homogenization at 25 °C (NH+Homo-W); (3) continuous agitation at 80 °C (H as a control); (4) homogenization before *T_p_* at 80 °C (H+Homo-BG); (5) homogenization after *T_p_* at 80 °C (H+Homo-AG); (6) homogenization after *T_o_* at 80 °C (H+Homo-G); (7) continuous homogenization at 80 °C (H+Homo-W). Agitation was performed at 250 rpm, and the homogenization speed was 10,000 rpm. For H+Homo-BG (4), H+Homo-AG (5), and H+Homo-G (6), the groups were continuously agitated at 250 rpm for the remaining time instead of homogenization. Each 300 mL carbohydrate-binder mixture was placed in a glass beaker and heated in a water bath, in which the agitation (C-STMD15, Changshin Science, Seoul, Republic of Korea) or homogenization (T18 digital ULTRA TURRAX^®^, IKA Works, Inc., Staufen, Germany) was performed. All treatments were completed within 16 min.

After the selection of the treatment era, the changes in the viscosity of the carbohydrate-binder mixture and the compression strength of the FD binder block according to the homogenization speed (0–10,000 rpm) and concentration of the carbohydrate-binder mixture (7.25–25.38%) were examined to find a suitable condition for the preparation of the FD binder block.

### 2.5. Determination of the Core Temperature and Viscosity

The change in the core temperature of the carbohydrate-binder mixture was monitored every 30 s using a thermo-hygrometer (RC-4HC, Elitech Inc., Mailpitas, CA, USA). The change in the viscosity of the carbohydrate-binder mixture was measured at a speed of 30 rpm using a Brookfield viscometer (RV-DV Ⅱ Pro+, Brookfield Engineering Inc., Middleboro, MA, USA) with a spindle No. S62 (diameter: 18.7 mm, height: 6.9 mm). To minimize the influence of homogenization and agitation during viscosity measurement, homogenization and agitation were stopped for 5 s to stabilize before measurement, and the viscosity was measured and expressed in cP units.

### 2.6. Freeze-Drying Condition

Three hundred milliliters of each sample was poured into a polypropylene square container (3.0 cm × 3.0 cm × 1.5 cm; width, length, and height, respectively). The sample was frozen at −50 °C for 24 h, then freeze-dried with the application of Donglimfood Co. Ltd. (Gangneung, Republic of Korea) at an ice condenser temperature of −50 °C and pressure of 0.67 Pa for 72 h. The FD binder block samples were sealed in polyethylene bags and stored at room temperature; the relative humidity was maintained at 40% for further experiments.

### 2.7. Observation of Appearances of the Binder Mixture and Its FD Block

The appearances of the binder mixture in the molding stage and the FD block were photographed using a phone camera (iphone SE2, Apple Inc., Cupertino, CA, USA).

### 2.8. Measurement of Compression Strength of the FD Binder Block

The compression strength of the FD binder block was measured using a texture analyzer (TA-XT plus, Stable Micro Systems Inc., Godalming Surrey, UK), as described by Harnkarnsujarit et al. [18] with some modifications. A circular-type probe (P/36R, diameter = 35 mm) was used to measure the compression strength, and the sample was measured in the compression mode at a measurement speed of 2 mm/s. When the penetration depth was set to approximately 66%, the FD sample was completely damaged.

### 2.9. Scanning Electron Microscopy

The microstructure of the FD binder block was observed using a scanning electron microscope (SEM; Nanoeye, SNE-3000M; SEC Co., Ltd., Suwon, Republic of Korea), as described by Lee et al. [19]. The surface sample (1.5 cm × 1.5 cm × 0.5 cm; width, length, and thickness) was collected by cutting using a razor blade. After platinum coating, the samples were observed at ×50 and ×500 magnifications, and at 30 kV.

### 2.10. Measurement of Dispersion Stability of the FD Binder Block after Rehydration

The dispersion stability of the FD binder block was measured using Turbiscan (Turbiscan Lab, Formulaction Scientific Instruments, Toulouse, France). One FD binder block (approximately 3.5 g per 1 block) was suspended in 200 mL water at 80 °C and dispersed using a glass stick. The suspended liquid was transferred into a turbiscan vial (25 mm in diameter; 50 mm in height) and the transmission value (T%) was measured at 50 °C for 1 h with 30 s interval.

### 2.11. Data Analysis

All values are expressed as the mean ± standard deviation (SD) in triplicate determinations. The thermal properties, viscosities, and compression strengths were statistically assessed using the IBM SPSS Statistics program 20 (IBM, Armonk, NY, USA), and significant differences (*p* < 0.05) in the means were identified using Tukey’s test.

## 3. Results and Discussion

### 3.1. Thermal Properties of Carbohydrate-Binders and Their Mixtures on the DSC

Maltodextrin, potato starch, and rice flour were used as the carbohydrate-binders in this study, and their thermal properties were profiled using DSC (Table 2). DSC measures the heat flow associated with structural changes depending on time and temperature. When heated in the presence of water, the intermolecular bonds within starch granules break down, which is called gelatinization [20]. Gelatinization of potato starch and rice flour was initiated at 61.4 and 58.7 °C (*T_o_*), reached the peaks at 66.3 and 70.2 °C (*T_p_*), and then terminated at 74.9 and 79.8 °C (*T_c_*), respectively. Maltodextrin is a hydrolysate of starch that does not retain the original molecular organization of its original starch; therefore, it did not present gelatinization phenomena in the DSC analysis.

The carbohydrate-binder mixture was composed of maltodextrin, potato starch, and rice flour in a ratio of 1:0.25:0.2. The gelatinization enthalpy of the mixture was lower than that of potato starch and rice flour because the mixture contained significantly lower amounts of potato starch and rice flour, which are the sources of melting enthalpy in the sample. The onset (64.8 °C), peak (71.2 °C), and completion (81.0 °C) temperatures were higher in the mixture. This was likely due to interactions occurring during mixing and competition for water caused by the high moisture affinity of maltodextrin, which influenced the increase in the temperature range. This corresponds with a previous study by Pourmohammadi et al. [21], who reported an increase in the gelatinization temperature of starch affected by maltodextrin. In the present study, the physical treatment eras were diversified based on when the core temperature reached 71 °C, at which the gelatinization of the binder mixture was expected on the DSC results. The gelatinization of starch leads to the leaching and subsequent arrangement of starchy molecules, which significantly affects to the stability of the network [20]. High-speed homogenization was considered to ensure the homogeneity of the FD binder block. By investigating the effects of physical treatment before, during, and after gelatinization, we aimed to suggest suitable conditions to enhance the homogeneity and overall quality of FD soup products with improving process efficiency.

### 3.2. Effects of Homogenization Era on the Viscosity of Carbohydrate-Binder Mixture

**Figure 1:**
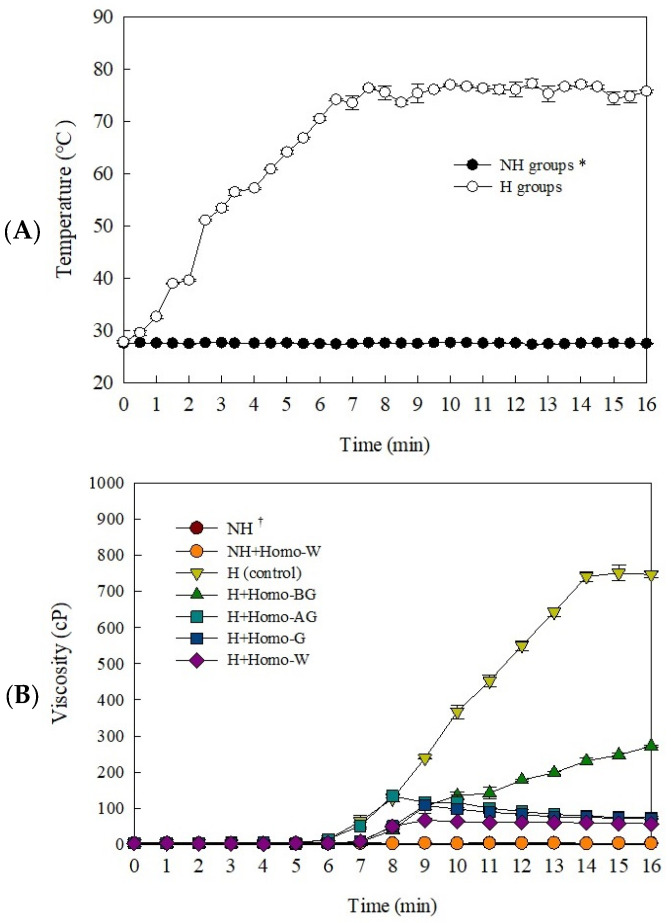
Changes in the core temperature (**A**) and viscosity (**B**) of the carbohydrate-binder mixture according to the homogenization era during gelatinization. * NH group, without heat treatment; H group, with heat treatment. ^†^ NH, continuous agitation at 25 °C; NH+Homo-W, continuous homogenization at 25 °C; H, continuous agitation at 80 °C; H+Homo-BG, homogenization before *T_o_* at 80 °C (0–7 min); H+Homo-AG, homogenization after *T_p_* at 80 °C (8–15 min); H+Homo-G, homogenization from reaching *T_o_* at 80 °C (5–12 min); H+Homo-W, continuous homogenization at 80 °C (0–16 min). Data expressed as the mean ± SD of triplicate experiments.

The preparation process for block-type FD soup products includes hydrothermal mixing of the binder, source base, and major and minor ingredients, with a heat treatment time of almost within 15–20 min to allow the carbohydrate-binders to be gelatinized [9]. Figure 1 shows the changes in the core temperature and viscosity of the carbohydrate-binder mixture according to the homogenization era (Table 1). The core temperatures of the carbohydrate-binder mixture in the non-heat-treated groups were maintained at approximately 27.5 °C during the whole treatment time, whereas the core temperature in the heat-treated groups constantly increased to approximately 77.0 °C at 7.5 min and then was maintained until 16.0 min (Figure 1A). The temperature of the carbohydrate-binder mixture reached the onset temperatures (*T_o_*, 64.8 °C) at 5.0 min and the peak temperature (*T_p_*, 71.2 °C) was attained at 6.5 min.

In the present study, the viscosities of the two non-heated groups were maintained around 3.0 cP throughout the treatment period (Figure 1B). Maltodextrin (DE 28) is soluble and does not exhibit viscosity regardless of temperature. Therefore, the viscosity of this binder is likely influenced by the starchy materials, potato starch, and rice flour. Starch needs to gelatinize to develop viscosity, but since starch does not gelatinize at room temperature, no viscosity was observed in the NH group. The viscosity of group H (control, with heating and continuous agitation at 250 rpm) increased rapidly from 7.0 min to 14.0 min, reached 741.0 cP, and was maintained until 16.0 min. By the homogenization era, the developmental aspect of the viscosity was different; the viscosity of the H+Homo-AG group increased similarly to that of the control until 8.0 min and then decreased until 74.0 cP with homogenization (8.0–16.0 min), whereas that of the H-Homo-BG group increased slowly from 8.0 min (45.0 cP) to 16.0 min (269.9 cP). For H+Homo-G, the viscosity peaked at 9.0 min (107.5 cP) and then slowly decreased. The viscosity of H+Homo-W was lower than the other homogenized groups.

The homogenization treatment reduced the viscosity of the carbohydrate-binder mixture regardless of the treatment era, and homogenization around *T_0_* retarded the formation of viscosity. During the gelatinization of starch, granular swelling, loss of molecular order and crystallinity, water intake, an increase in viscosity, and amylose solubilization occur [22]. Hydrothermal agitation at an appropriate rate during gelatinization improves the collapse of granules and amylose elution, which increases viscosity and gel formation, whereas the high-speed shear rate interferes with starch rearrangement, decreasing viscosity [23,24]. Physical treatments can alter the rheological behavior of starches [16], and a decrease in the viscosity of starch when combined with heat and high-speed shearing treatment has been reported in other studies [24]. During starch gelatinization, amylose is eluted after the collapse of starch granules, and viscosity is displayed. The decrease in the viscosity of the binder mixture by homogenization may be a phenomenon in which starchy molecules are not randomly arranged owing to the high-speed shear force and are regularly aligned in one direction [20,23,24].

### 3.3. Appearances of the Carbohydrate-Binder Mixtures during Molding Process and Their FD Binder Blocks

**Figure 2:**
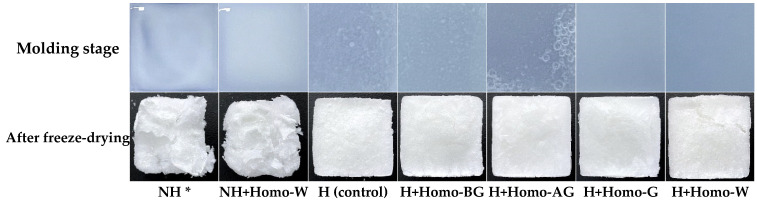
Appearance of the carbohydrate-binder mixtures during the molding process and their FD binder blocks. * NH, continuous agitation at 25 °C; NH+Homo-W, continuous homogenization at 25 °C; H, continuous agitation at 80 °C; H+Homo-BG, homogenization before *T_p_* at 80 °C (0–7 min); H+Homo-AG, homogenization after *T_p_* at 80 °C (8–15 min); H+Homo-G, homogenization from reaching *T_o_* at 80 °C (5–12 min); H+Homo-W, continuous homogenization at 80 °C (0–16 min).

The appearance of the carbohydrate-binder mixtures during the molding process and their FD binder blocks are shown in Figure 2. The binder mixtures in the NH group appear to be in the form of a dispersion of carbohydrate particles. Regarding the heat-treated groups, starch agglomeration was observed in the carbohydrate-binder mixtures of the H and H+Homo-BG groups during the molding process. Air bubbles were produced by homogenization but most disappeared as gelatinization progressed. However, they existed only in the H+Homo-AG group, were retained until the molding process, and negatively affected the surface of the FD binder block, indicating that it is not desirable to start the homogenization treatment after reaching *T_p_*. The surface of the FD blocks was similar to that of the molding process; the surface of the H group was rough, whereas those of the homogenized groups, except for the H+Homo-BG group, were smooth; however, a crack in the FD binder block occurred in the H+Homo-W group, indicating that excessive homogenization had a negative effect on the structural stability of the FD binder block.

### 3.4. Effect of Homogenization Era on the Compression Strength of the FD Binder Block

**Figure 3:**
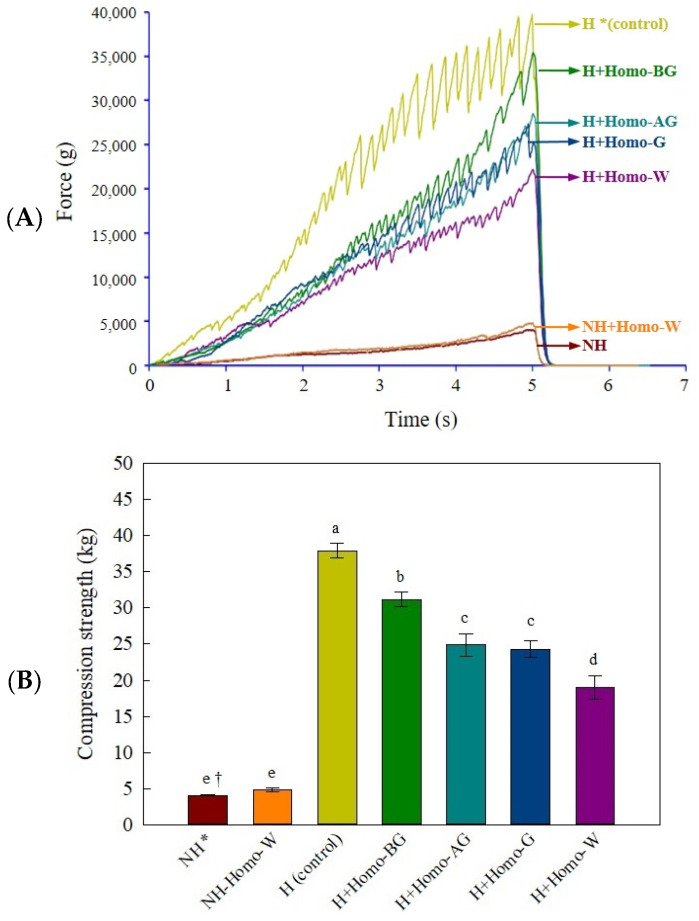
Breakdown profiles (**A**) and compression strengths (**B**) of carbohydrate-binder mixtures according to homogenization era during gelatinization. * NH, continuous agitation at 25 °C; NH+Homo-W, continuous homogenization at 25 °C; H, continuous agitation at 80 °C; H+Homo-BG, homogenization before *T_p_* at 80 °C (0–7 min); H+Homo-AG, homogenization after *T_p_* at 80 °C (8–15 min); H+Homo-G, homogenization from reaching *T_o_* at 80 °C (5–12 min); H+Homo-W, continuous homogenization at 80 °C (0–16 min). Data expressed as the mean ± SD of triplicate experiments. ^†^ The different letters indicate significantly different values (*p* < 0.05).

The molecular structure of carbohydrates is a critical intrinsic factor that can significantly affect starch gelatinization properties [25], and the network structures of gelatinized binder mixtures may alter the ice habits (size, number, and morphology) during freezing [26]. The compression strength of the FD binder block was measured to evaluate its structural stability. In terms of the stress force and compression strength of the FD binder block (Figure 3), the heated groups were significantly higher than the non-heated groups (*p* < 0.05), which failed to maintain their block shape after freeze-drying. The compression strength of the FD blocks tended to increase with increasing viscosity of the carbohydrate-binder mixtures. Among the heated groups, the compression strength was highest in the H group, followed by H+Homo-BG, H+Homo-AG, H+Homo-G, and H+Homo-W. As mentioned in the appearance results, the surface of the FD binder block in the heated-homogenized groups, except for H+Homo-BG, was fine and smooth compared to that of the H group. However, a crack on the surface of the FD binder block occurred in the H+Homo-W group, and the block of H+Homo-AG had an uneven surface. Thus, H+Homo-G was selected as a suitable homogenization era for the subsequent experiments.

### 3.5. Effects of Homogenization Speed and Mixture Concentration on the Viscosity and Compression Strength of the FD Binder Block

**Figure 4:**
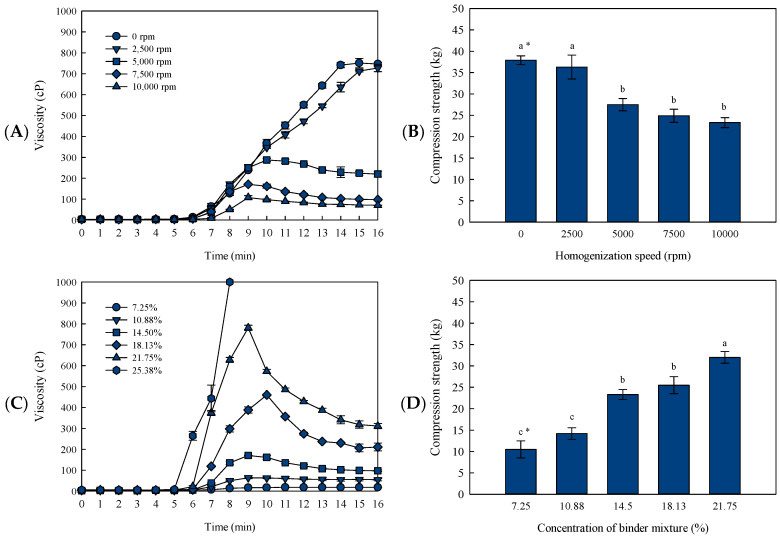
Changes in the viscosities of the carbohydrate-binder mixtures and compression strengths of the FD binder blocks according to the homogenization speed and mixture concentration. The viscosity of the binder mixture according to the homogenized speed, (**A**); the compression strength of the FD binder blocks according to the homogenization speed, (**B**); the viscosity of the binder mixture according to the mixture concentration, (**C**); the compression strength of the FD binder block according to the mixture concentration, (**D**). Data expressed as the mean ± SD. of triplicate experiments. * Different letters indicate significantly different values (*p* < 0.05).

To find a suitable condition for the preparation of the FD binder block, the effects of the homogenization speed and concentration of the carbohydrate-binder mixture were examined. The treatment era was fixed as the H+Homo-G group (Figure 4). According to the homogenization speed, the viscosity of the carbohydrate-binder mixture and the compression strength of the FD binder block tended to decrease inversely with increasing speed when treated above 5000 rpm, but there was no significant difference in the compression strengths from 5000 to 10,000 rpm (*p* < 0.05). For manufacturing block-type commercial FD soup products, the concentration of the carbohydrate-binder mixture varies depending on the type of ingredients and the purpose of the product, and the mixture generally consists of 5–20% of the total mixing weight. In this study, the concentrations were tested in the range of 7.25% to 25.38%, with the homogenization speed fixed at 7500 rpm (Figure 4C, D). The viscosity of the carbohydrate-binder mixture and compression strength increased with increasing concentration. The viscosity of the mixture at 25.38% increased rapidly after 6.0 min, making it difficult to measure the viscosity after 8.0 min, whereas the mixtures at less than 10.88% exhibited much lower compression strengths than the others. Therefore, the concentration was suggested to range from 14.5% to 21.8% for preparing the FD binder block.

### 3.6. Surface Microstructure of the FD Binder Block

**Figure 5:**
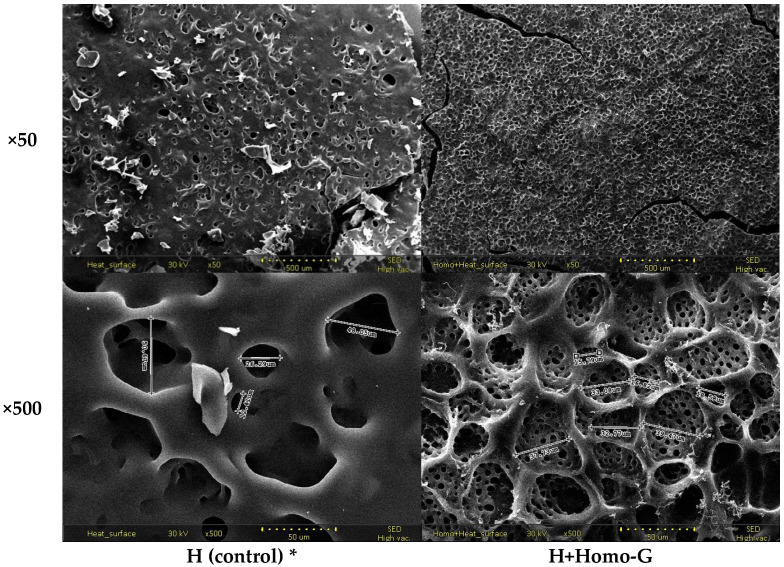
Surface microstructures of FD binder blocks with and without homogenization during gelatinization. * H, continuous agitation (250 rpm) at 80 °C; H+Homo-G, homogenization (7500 rpm) from reaching *T_o_* at 80 °C (5–12 min). SEM images were obtained at ×50 and ×500 magnifications with setting at a voltage of 30 kV.

The surface microstructures of the FD binder blocks of the H and H+Homo-G groups are shown in Figure 5. The SEM images revealed the interconnected network structure of the binder materials and embedded pores formed by water-ice crystals during the pre-freezing step. While huge pores sparsely appeared on the surface of the H group (12.4–50.7 µm in diameter), more homogeneously huge pores (15.8–39.5 μm in diameter) with tiny regular pores (less than 2 μm) appeared on the surface of the H+Homo-G group.

The homogenization treatment of the carbohydrate-binder mixture applied in this study may accelerate the alignment of starches eluted from potato starch and rice flour, as stated above. This process can rapidly induce a disordered phase transition, resulting in irreversible disruption of starch semi-crystalline structures [27] and frequent generation of tiny pores. In addition, the presence of sugars such as maltodextrin applied in this study may affect the swelling behavior of starch by controlling the kinetics between the starch and water [28].

### 3.7. Dispersion Stability of the FD Binder Block after Rehydration

**Figure 6:**
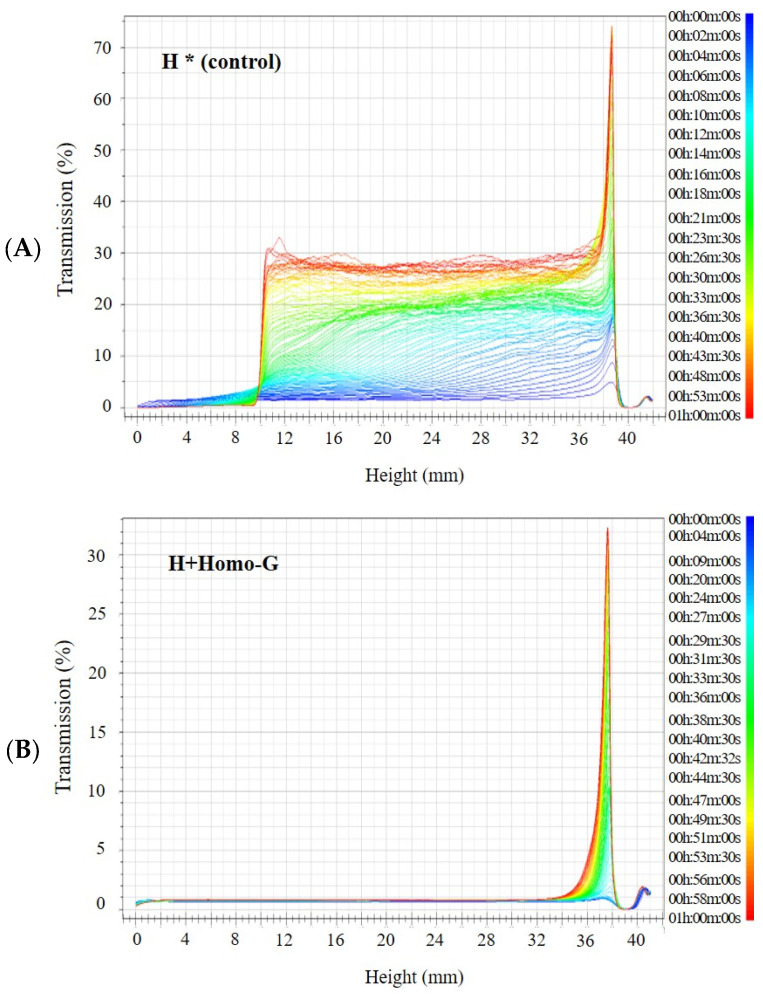
Change in transmission value of the rehydrated FD binder block after 1 h. * H, continuous agitation (250 rpm) at 80 °C, (**A**); H+Homo-G, homogenization (7500 rpm) from reaching *T_o_* at 80 °C (**B**).

The porosity of the FD blocks affects their rehydration capacity, which is important for reconstituted foods [18]. The dispersion stability after rehydration of the FD binder block of the H+Homo-G group (homogenization at 7500 rpm and 14.5% mixture concentration) was compared with that of the FD binder block of the H group (agitation at 250 rpm and 14.5% mixture concentration) on a Turbiscan (Figure 6). The transmission values in the two groups were similar (2%) immediately after rehydration. However, as the standing time elapsed, the transmission value of the H+Homo-G group was maintained, whereas that of the H group was divided into two phases at a height of approximately 10 mm, and the transmission value of the upper layer increased. In the Turbiscan stability index (TSI), the H group increased as the standing time passed, indicating that the dispersion stability decreased, whereas the H+Homo-G group was maintained.

## 4. Conclusions

This study was conducted to improve the quality of block-type FD soup products by modifying the physical properties of a carbohydrate-binder mixture composed of malto-dextrin, potato starch, and rice flour via high-speed homogenization during hydrothermal gelatinization. The homogenization era, speed, and concentration of the mixture were considered as experimental conditions; in particular, the homogenization era was set by considering the hydrothermal property of the binder mixture profiled using DSC. Although the homogenization treatment reduced the viscosity of the binder mixture regardless of the treatment era during gelatinization, the quality of the FD binder block was improved by homogenization above 5000 rpm when the core temperature of the mixture reached approximately *T_o_* in the concentration of the mixture ranging from 14.5 to 21.8%. The improved FD binder block exhibited a fine surface and tiny porous microstructure compared with that of the control (with continuous agitation at 250 rpm). In terms of dispersion stability after rehydration, the control block was divided into two phases, whereas the improved block maintained the initial dispersion stability at 50 °C for 1 h. This approach can help improve the dispersion stability and reconstruction property after rehydration of FD soup products, including their appearance quality, which is expected to be referenced for the purpose of improving the quality of the FD soup products.

## Figures and Tables

**Table 1:** Experimental conditions for the preparation of carbohydrate-binder mixtures according to the treatment era.

Group *	Temperature (°C)	Homogenization	
		Speed (rpm)	Treatment Era (min)
NH	25	Agitation, 250	0–16
NH+Homo-W	25	10,000	0–16
H (control)	80	Agitation, 250	0–16
H+Homo-BG	80	10,000	0–7
H+Homo-AG	80	10,000	8–15
H+Homo-G	80	10,000	5–12
H+Homo-W	80	10,000	0–16

* NH, continuous agitation at 25 °C; NH+Homo-W, continuous homogenization at 25 °C; H, continuous agitation at 80 °C; H+Homo-BG, homogenization before *T_p_* at 80 °C; H+Homo-AG, homogenization after *T_p_* at 80 °C; H+Homo-G, homogenization from reaching *T_o_* at 80 °C; H+Homo-W, continuous homogenization at 80 °C. Maltodextrin (10 g), potato starch (2.5 g), and rice flour (2 g) were suspended in 100 mL of deionized water as a carbohydrate-binder mixture.

**Table 2:** Thermal properties of carbohydrate-binders and their mixtures.

Carbohydrate-Binder	Parameter *			
	*T_o_* (°C)	*T_p_* (°C)	*T_c_* (°C)	∆*H* (J/g)
Maltodextrin	Nd ^†^	Nd	Nd	Nd
Potato starch	61.4 ± 0.1 ^b,‡^	66.3 ± 0.4 ^c^	74.9 ± 0.6 ^b^	15.5 ± 0.5 ^a^
Rice flour	58.7 ± 0.5 ^c^	70.2 ± 0.2 ^b^	79.8 ± 0.1 ^a^	8.9 ± 0.4 ^b^
Mixture	64.8 ± 0.5 ^a^	71.2 ± 0.3 ^a^	81.0 ± 1.8 ^a^	4.2 ± 0.9 ^c^

* *T_o_*, onset temperature; *T_p_*, peak temperature; *T_c_*, completion temperature; ∆*H*, gelatinization enthalpy. ^†^ Not detected. Data expressed as the mean ± SD. of triplicate experiments. ^‡^ The different letters indicate significantly different values within the carbohydrate types (*p* < 0.05).

## Data Availability

The original contributions presented in the study are included in the article, further inquiries can be directed to the corresponding authors.
